# Automated basal insulin delivery versus multiple daily injections in type 1 diabetes: results from a randomized parallel controlled trial

**DOI:** 10.3389/fendo.2025.1716587

**Published:** 2025-12-19

**Authors:** Johan H. Jendle, Satish K. Garg, Charles Thivolet, Ruth S. Weinstock, Irl B. Hirsch, Mark Evans, Kurt J. Griffin, Athena Philis-Tsimikas, Benjamin J. Wheeler, Mark S. Kipnes, Anders L. Carlson, Bruce A. Buckingham, Anuj Bhargava, Bruce W. Bode, Margaret L. Lawson, Amy B. Criego, Janet B. McGill, John “Chip” H. Reed, Gnanagurudasan Prakasam, George Grunberger, Angela Girelli, María Asunción Martinez-Brocca, Mark P. Christiansen, Martin I. de Bock, Yogish C. Kudva, Scott W. Lee, Caroline Yovanovich, John J. Shin, Toni L. Cordero, Jennifer J. F. McVean, Robert A. Vigersky

**Affiliations:** 1 School of Medicine, Department of Medical Sciences, Örebro University, Örebro, Sweden; 2 Barbara Davis Center for Diabetes, University of Colorado- Denver, Aurora, CO, United States; 3 Hybrid Closed-Loop (HCL)- Diab-eCare, Lyon, France; 4 Endocrinology, Diabetes and Metabolism, SUNY Upstate Medical University, Syracuse, NY, United States; 5 Division of Metabolism, Endocrinology and Nutrition, University of Washington School of Medicine, Seattle, WA, United States; 6 Institute of Metabolic Science and Department of Medicine, University of Cambridge, Cambridge, United Kingdom; 7 Sanford Research, Sioux Falls, SD, United States; 8 Department of Endocrinology, Diabetes and Metabolism, Scripps Whittier Diabetes Institute, La Jolla, CA, United States; 9 Health New Zealand – Southern, Dunedin, New Zealand; 10 Diabetes and Glandular Disease Clinic, P.A., San Antonio, TX, United States; 11 Park Nicollet International Diabetes Center/Health Partners Institute, Minneapolis, MN, United States; 12 Stanford University School of Medicine, Stanford, CA, United States; 13 Iowa Diabetes Research, West Des Moines, IA, United States; 14 Atlanta Diabetes Associates, Atlanta, GA, United States; 15 Children’s Hospital of Eastern Ontario (CHEO), Ottawa, ON, Canada; 16 Division of Endocrinology, Metabolism and Lipid Research, Washington University - Saint Louis, St. Louis, MO, United States; 17 Endocrine Research Solutions, Roswell, GA, United States; 18 Sutter Children’s Center and Sutter Medical Group, Sacramento, CA, United States; 19 Grunberger Diabetes and Endocrinology, Bloomfield Hills, MI, United States; 20 Azienda Ospedaliera Spedali Civili di Brescia, Brescia, Italy; 21 Department of Endocrinology and Nutrition, Hospital Universitario Virgen Macarena, Sevilla, Spain; 22 Flourish Walnut Creek, Walnut Creek, CA, United States; 23 Department of Paediatrics and Child Health, New Zealand Clinical Research, University of Otago, Christchurch, New Zealand; 24 Department of Endocrinology, Mayo Clinic Rochester, Rochester, MN, United States; 25 Medtronic Diabetes, Northridge, CA, United States

**Keywords:** type 1 diabetes, automated insulin delivery, HbA1c, time below range, time in range, diabetes treatment satisfaction, adult, pediatric

## Abstract

**Introduction:**

This study evaluated 6-month effectiveness and safety of automated insulin delivery (AID) in comparison with multiple daily injections (MDI) in pediatric and adult type 1 diabetes (T1D).

**Materials and methods:**

Individuals with T1D, aged 2–80 years, were enrolled across 32 international centers (in the United States, Europe, Canada, and New Zealand) and randomized 1:1 to AID intervention (MiniMed™ 670G or 770G system) or MDI with or without continuous glucose monitoring. Primary endpoints were change in mean HbA1c for participants with a baseline HbA1c >8.0% (Group 1) and percentage of time spent below 70 mg/dL (%TBR <70 mg/dL [<3.9 mmol/L]) for participants with baseline HbA1c ≤8.0% (Group 2), to show superiority of AID intervention versus MDI. Safety endpoints including rates of severe hypoglycemia and diabetic ketoacidosis (DKA), and difference in diabetes treatment satisfaction score were assessed.

**Results:**

For Group 1, N = 56 participants (aged 29.4 ± 17.0 years) were randomized to AID intervention and N = 54 participants (aged 36.8 ± 19.6 years) were randomized to MDI. For Group 2, N = 73 (aged 37.4 ± 21.0 years) and N = 69 (aged 39.2 ± 19.3 years), respectively, were randomized to AID and MDI. Change in HbA1c (mean [95% CI] difference of −0.7% [−1.1, −0.3], P = 0.0002) and difference in %TBR <70 mg/dL (4.8% [−6.4, −3.1], P<0.001) favored AID intervention versus MDI. Rates of severe hypoglycemia (AID: 1.82/100 patient-years) and DKA (MDI: 3.52/100 patient-years) were low and met preestablished success criteria for safety.

**Discussion:**

This large, international, multicenter randomized controlled trial demonstrates safety of the MiniMed™ 670G/770G systems. AID significantly improved HbA1c and time spent in hypoglycemia when compared with MDI, in both youth and adults living with T1D.

**Clinical trial registration:**

## Introduction

The majority of international guidelines recommend automated insulin delivery (AID) systems as the standard of care for people living with type 1 diabetes (T1D), both for adult and pediatric populations (1, 2). Systematic reviews and meta-analyses have demonstrated the superiority of AID versus multiple daily injections (MDI) or continuous subcutaneous insulin infusion (CSII) with or without continuous glucose monitoring (CGM) (3–5), through an improved glycated hemoglobin (HbA1c) and percentage of time in range (%TIR), and a concomitant reduction in or unchanged time below range (%TBR <70 mg/dL [<3.9 mmol/L]).

The Medtronic MiniMed™ 670G (MM670G) system was the first AID system approved for diabetes care (September 28, 2016). It used a proportional integrated derivative-insulin feedback module algorithm that provides adaptive basal AID every 5 min based on current and predicted sensor glucose (SG) readings and a preset glucose target (e.g., 120 mg/dL [6.7 mmol/L], or 150 mg/dL [8.3 mmol/L], if using the temporary target). As part of the new Medtronic AID device family equipped with telemetry, the MiniMed™ 770G (MM770G) system, which has the same algorithm as the MM670G system, was approved August 31, 2020, and designed to automatically upload and display real-time insulin delivery metrics and CGM data directly through the MiniMed™ mobile app by Bluetooth™ technology.

In 2023, Garg and colleagues published results from the multicenter, adaptive, randomized controlled trial (RCT) evaluating MM670G AID versus continuous subcutaneous insulin infusion (CSII) control and demonstrated safety with significantly improved HbA1c in favor of AID intervention, irrespective of baseline HbA1c glycemic control, in pediatric and adult T1D (6). The present study reports findings from a parallel RCT evaluating MM670G/770G AID versus MDI with and without CGM, in pediatric and adult T1D.

## Materials and methods

### Study design

The *Multicenter Trial in Adult and Pediatric Patients with T1D Using a Hybrid Closed-Loop (HCL) System and Control at Home* Trial (ClinicalTrials.gov NCT02748018) comprised three separate randomized, parallel, adaptive study evaluations, to assess the safety and effectiveness of the MM670G/770G AID systems in participants with T1D aged 2–80 years. Each RCT evaluation included a 6-month comparator of multiple daily injections (MDI) with or without continuous glucose monitoring (CGM) and continuous subcutaneous insulin infusion (CSII) without CGM or sensor-augmented pump (SAP) control. Study enrollment for the CSII evaluation (for which results have been published) (6) occurred at 23 sites across the United States (N = 22) and Canada (N = 1). Enrollment for the present study (AID versus MDI evaluation) was conducted at 32 sites across the United States (N = 22), Canada (N = 2), New Zealand (N = 2), and Europe (N = 6) and participants randomized to the AID intervention arm used either a MiniMed™ 670G (MM670G) system or MiniMed™ 770G (MM770G) system.

The trial complied with the Declaration of Helsinki and, where applicable, the United States Food and Drug Administration Code of Federal Regulations Title 21, Health Canada Regulations (SOR/98-282), the International Organization for Standardization (ISO 14155:2011), and applicable laws and requirements (national and local). The protocol was approved by the central Internal Review Board (IRB) Advarra (formerly Quorum IRB) and local investigational site IRBs. Informed consent and assent, when applicable, were obtained before study start.

### Participants

Individuals (2–80 years of age) with a clinical diagnosis of T1D and who used MDI with or without CGM for ≥3 months before screening were included. To participate successfully, the following were required: a total daily insulin dose of ≥8 units/day and the ability to perform or reliably undergo ≥4 blood glucose measurements (BGMs) per day. Individuals aged 2–21 years were required to have requisite support. Exclusion criteria included participation in a previous closed-loop device study; inability to tolerate tape adhesive around the glucose sensor; and any unresolved adverse skin condition around the glucose sensor or transcutaneous infusion set. The full list of eligibility criteria is in **Supplementary Material S1**. All participants provided written informed consent or assent (when applicable) before starting the study.

### Study schedule and randomization

The study visit schedule is listed in **Supplementary Material S2**, and visit 1 included bloodwork for a central laboratory HbA1c test, in addition to completion of the Diabetes Treatment Satisfaction Questionnaire status (DTSQs) form. All participants underwent masked CGM (Guardian™ Sensor 3 glucose sensor connected to the Guardian™ Link transmitter [Medtronic]) during a run-in period of 2 weeks (visits 1-3) and were expected to demonstrate appropriate glucose sensor wear and perform requested daily BGMs with a CONTOUR^®^NEXT LINK 2.4 blood glucose meter (Ascensia Diabetes Care, Parsippany, NJ) or Accu-Chek^®^ Guide Link blood glucose meter (Roche Diabetes Care, Inc., Indianapolis, IN), which were used for sensor calibration. The CGM data captured during run-in provided baseline metrics for both the AID intervention and control arm.

At the end of the run-in period, participants underwent computer-generated 1:1 randomization into AID intervention or control and were stratified based on their baseline HbA1c such that Group 1 comprised participants with baseline HbA1c >8.0% and Group 2 included only those with baseline HbA1c ≤8.0%. Participants 2–6 years of age were automatically entered into the AID intervention arm. At the beginning of the study period (visit 4), the AID intervention arm started CGM and enabled Auto Mode for 6 months. For the control arm, preexisting diabetes management therapy was continued for 6 months. Follow-up office and telephone visits occurred until the end of the study period (visit 9), when bloodwork for a central laboratory HbA1c test was collected, and the DTSQs and Diabetes Treatment Satisfaction Questionnaire change (DTSQc) were completed.

### System settings

For the AID intervention arm, the study pump was set with the automated basal glucose target (GT) of 120 mg/dL (6.7 mmol/L) and allowed a temporary target of 150 mg/dL (8.3 mmol/L). It was recommended to set the high SG limit alert at 300 mg/dL and the low SG limit alert at 70 mg/dL (3.9 mmol/L). For participants 2–6 years of age, the low SG alert was advised to be no lower than 70 mg/dL. The insulin-to-carbohydrate ratios and active insulin time were adjusted as needed and based on the investigator’s discretion. For the MDI control arm, insulin therapy and adjustments were as needed and based on the investigator’s discretion.

### Primary and secondary endpoints

For Group 1 (baseline HbA1c >8%), the primary endpoint was the difference in HbA1c change from baseline to the end of the 6-month study period. The goal was to show superiority of the AID intervention arm compared with the control arm in HbA1c reduction. For Group 2 (baseline HbA1c ≤8%), the primary endpoint was the difference in the percentage of time spent below 70 mg/dL (%TBR <70 mg/dL). The goal was to show superiority of the AID intervention arm compared with the control arm in reducing time in hypoglycemia. The secondary endpoint for Group 1 was the difference in %TBR <70 mg/dL to show non-inferiority of the AID intervention arm compared with the control arm in reducing time in the hypoglycemic range. For Group 2, the secondary endpoint was the difference in HbA1c change from baseline to the end of the 6-month study period to show noninferiority of the AID intervention arm compared with the control arm in reducing HbA1c.

For the primary effectiveness endpoints, the sample size estimates for change in HbA1c were based on a two-sample *t* test with a one-sided type I error rate of 2.5%. For Group 1, assuming a mean change in HbA1c of −0.45% in the AID arm and of −0.1% in the MDI control arm (with a standard deviation of 0.5% for both arms), a power and sample size calculator showed that a total of 140 participants (N = 70 in AID and N = 70 in MDI control) would provide over 95% power to detect the superiority of the AID arm. The sample size estimates for %TBR <70 mg/dL were, also, based on a two-sample *t* test with a one-sided type I error rate of 2.5%. For Group 2 and assuming an AID arm mean %TBR <70 mg/dL of 5% with a standard deviation of 4% and an MDI control arm mean %TBR <70 mg/dL of 9% with a standard deviation of 6%, a total of 140 participants (N = 70 in AID and N = 70 in MDI control) would provide over 90% power to detect the superiority of the AID arm. The same *t* test and type I error rate estimated similar sample sizes (N = 70 in AID and N = 70 in MDI control) to provide over 90% power to detect non-inferiority of the AID arm compared with the MDI control arm, for the secondary effectiveness endpoints. For Group 1, a mean %TBR <70 mg/dL of 4% with a standard deviation of 3% was assumed for the AID arm and a mean %TBR <70 mg/dL of 6% with a standard deviation of 4% was assumed for the MDI control arm (non-inferiority margin of 2%). For Group 2, a mean change in HbA1c of −0.1% was assumed for the AID arm and of 0.0% in the MDI control arm, and a standard deviation of 0.7% for both arms (non-inferiority margin of 0.4%).

### Additional endpoints

Additional key CGM-derived glycemic endpoints for Group 1 and Group 2 of the AID intervention arm and control arm were compared and included mean SG, SD of SG, coefficient of variation (CV) of SG, and percentage of time spent at additional SG ranges (i.e., <54 mg/dL [<3.0 mmol/L], <70 mg/dL, 70–180 mg/dL [3.9-10.0 mmol/L], >180 mg/dL, and >250 mg/dL [>13.9 mmol/L]). For the control arm, 2-week masked CGM at specific timepoints post-randomization was conducted in parallel with the intervention arm using the AID device. The same endpoints were also assessed in an exploratory glycemic metrics analysis of participants aged 2–17 and 18–80 years.

### Participant-reported outcomes

Self-reported responses to the DTSQs were collected during baseline and at the end of the study and asked participants to rank treatment satisfaction on a 7-point scale from “very dissatisfied” to “very satisfied”. Queries were based on the sum of six items (current treatment satisfaction, convenience, flexibility, understanding of diabetes, treatment recommendation to others, and willingness to continue treatment), whereas two additional items related to the perception of high and low blood glucose control. Responses to the DTSQc were captured at the end of the study only. The scores for total diabetes treatment satisfaction (ranging from −36 to +36 for the DTSQs and from −18 to +18 for the DTSQc) were based on the sum of six items (current treatment satisfaction, convenience, flexibility, understanding of diabetes, treatment recommendation to others, and willingness to continue treatment) ranked on a 7-point scale from “much less [ … ] now” to “much more [ … ] now”. In addition to the perceived hypoglycemia and hyperglycemia items (scores ranging from −3 to +3), the DTSQc asked participants to compare their current diabetes treatment with their diabetes treatment before the study started.

### Safety endpoints

The primary safety endpoints were reported for each randomized cohort and were based on event rate (100 patient-years) including severe hypoglycemia (defined as an event requiring the active assistance of another individual to administer carbohydrate, glucagon, or other resuscitative actions due to altered participant consciousness) and DKA (defined as blood glucose >250 mg/dL, arterial pH <7.3, bicarbonate <15 mEq/L, and moderate ketonuria or ketonemia, requiring treatment within a health care facility). In addition, serious adverse events (SAEs), serious adverse device effects (SADEs), unanticipated adverse device effects (UADEs), and deaths were reported.

### Statistical and descriptive analyses

The primary effectiveness endpoint for Group 1 (baseline HbA1c >8.0%) underwent hierarchical analyses to determine the superiority of the AID intervention compared with control in HbA1c reduction and, for Group 2 (baseline HbA1c ≤8%), the superiority of the AID intervention compared with control in %TBR <70 mg/dL reduction. The secondary effectiveness endpoint for Group 1 included determining noninferiority of the AID intervention compared with control in %TBR <70 mg/dL reduction and, for Group 2, HbA1c reduction.

For primary and secondary endpoints involving HbA1c, a multiple imputation (MI) was applied for missing HbA1c data using an imputation regression method (*yˆ* + *z^r*), where *yˆ* is the predicted value, *z* is a standard normal random variable, and *^r* is the estimated standard deviation (SD) from the regression model. Age, sex, baseline HbA1c, diabetes duration, and BMI were independent variables in the model. Imputations were conducted five times using the MI procedure, and results were combined to form one inference using the MIANALYZE procedure in SAS™ 9.4 (SAS Institute, Cary, NC).

Comparisons of mean [95% confidence interval] difference in HbA1c change and difference in %TBR<70 mg/dL between AID intervention and control were conducted with one-way analysis of variance (ANOVA), and P < 0.05 was considered statistically significant. For additional key glycemic endpoints, the mean [95% CI] difference between AID intervention and control was determined. These glycemic endpoints underwent descriptive analysis by age group (2–17 years and 18–80 years). The mean [95% CI] of DTSQs and DTSQc scores were assessed to determine difference between AID intervention and control. Comparisons and analyses were conducted for both Group 1 and Group 2.

## Results

### Study participant disposition and baseline characteristics

**Figure 1:**
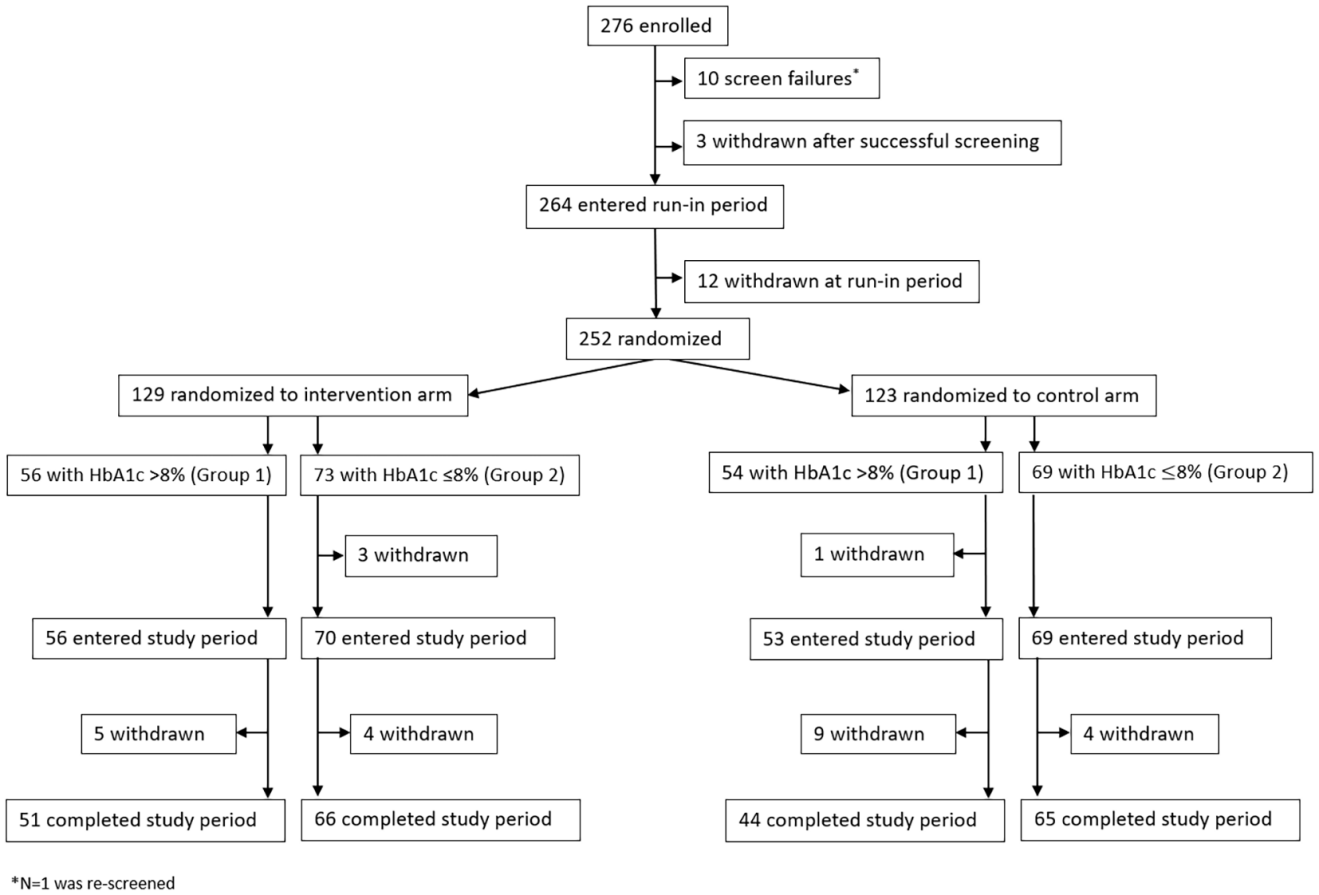
Study participant disposition from enrollment to completion.

A total of 276 individuals were enrolled in the study (**Figure 1**). There were 10 screen failures, 3 early withdrawals due to burden, time commitment, and personal reasons, and 12 withdrawals during the run period that were due to burden (N = 5), time commitment (N = 3), non-compliance (N = 1), personal reasons (N = 1), and an adverse event (N = 2). A total of 252 participants were randomized to either the AID intervention arm (N = 129; N = 56 in Group 1 and N = 73 in Group 2) or the MDI control arm (N = 123; N = 54 in Group 1 and N = 69 in Group 2). In the intervention arm, withdrawals (N = 12) were due to burden (N = 6), non-compliance (N = 1), investigator decision (N = 1), and an adverse event (N = 4), whereas withdrawals from the control arm (N = 14) were due to burden (N = 3), time commitment (N = 2), personal reasons (N = 4), loss to follow up (N = 3), and relocation (N = 2). The baseline demographics and characteristics of Group 1 (baseline HbA1c >8.0%) and Group 2 (baseline HbA1c ≤8.0%) randomized participants are shown in **Tables 1**, **2**, respectively. They are also listed by the overall group in **Supplementary Material S3**.

**Table 1:** Group 1 (baseline HbA1c >8.0%) demographics and baseline characteristics.

	AID (N=56)	MDI control (N=54)
Age, years		
Mean	29.4 (17.0)	36.8 (19.6)
Range (min, max)	4.0, 70.0	4.0, 72.0
Sex, N (%)		
Male	19 (33.9%)	20 (37.0%)
Female	37 (66.1%)	34 (63.0%)
Baseline HbA1c, %		
Mean	9.1 (0.9)	8.9 (1.1)
Range (min, max)	8.1, 11.6	8.1, 13.1
Diabetes history, years		
Mean	14.3 (11.6)	15.5 (12.7)
Range (min, max)	0.0, 48.0	0.0, 51.0
Weight (kg)	72.1 (26.9)	74.6 (24.4)
BMI (kg/m^2^)	25.3 (5.9)	26.3 (6.2)
Racial ancestry		
White	47 (83.9%)	44 (81.5%)
Asian	0 (0.0%)	1 (1.9%)
Indigenous/First Nations	0 (0.0%)	0 (0.0%)
Asian; White	0 (0.0%)	1 (1.9%)
Black African	2 (3.6%)	2 (3.7%)
Black African; White	0 (0.0%)	1 (1.9%)
Not reportable per local laws	5 (8.9%)	4 (7.4%)
Other	1 (1.8%)	1 (1.9%)
White; Other	1 (1.8%)	0 (0.0%)
Ethnicity		
Non-Hispanic/Latino	47 (83.9%)	42 (77.8%)
Hispanic/Latino	4 (7.1%)	8 (14.8%)
Not reportable per local law/regulation	5 (8.9%)	4 (7.4%)
Not reported	0 (0.0%)	0 (0.0%)

Data are presented as mean (SD) or N (%).

HbA1c, glycated hemoglobin; AID, automated insulin delivery; BMI, body mass index; SD, standard deviation.

**Table 2:** Group 2 (baseline HbA1c ≤8%) demographics and baseline characteristics.

	AID (N=73)	MDI control (N=69)
Age, years		
Mean	37.4 (21.0)	39.2 (19.3)
Range (min, max)	6.0, 75.0	8.0, 76.0
Sex, N (%)		
Male	20 (27.4%)	22 (31.9%)
Female	53 (72.6%)	47 (68.1%)
Baseline HbA1c, %		
Mean	7.1 (0.6)	7.1 (0.6)
Range (min, max)	5.5, 8.0	5.8, 8.0
Diabetes history, years		
Mean	18.3 (15.9)	19.4 (14.8)
Range (min, max)	0.0, 61.0	0.0, 60.0
Weight (kg)	72.3 (23.2)	77.4 (20.2)
BMI (kg/m^2^)	24.6 (5.8)	26.0 (6.2)
Racial ancestry		
White	60 (82.2%)	61 (88.4%)
Asian	0 (0.0%)	1 (1.4%)
Indigenous/First Nations	1 (1.4%)	0 (0.0%)
Asian; White	0 (0.0%)	0 (0.0%)
Black African	3 (4.1%)	0 (0.0%)
Black African; White	0 (0.0%)	0 (0.0%)
Not reportable per local laws	6 (8.2%)	7 (10.1%)
Other	3 (4.1%)	0 (0.0%)
White; Other	0 (0.0%)	0 (0.0%)
Ethnicity		
Non-Hispanic/Latino	65 (89.0%)	54 (78.3%)
Hispanic/Latino	2 (2.7%)	8 (11.6%)
Not reportable per local law/regulation	6 (8.2%)	6 (8.7%)
Not reported	0 (0.0%)	1 (1.4%)

Data are presented as mean (SD) or N (%).

HbA1c, glycated hemoglobin; AID, automated insulin delivery; BMI, body mass index; SD, standard deviation.

### Primary and secondary endpoints

By the end of the 6-month study period, the percentage of time spent in closed loop was 77.6% ± 20.6% and 85.3% ± 13.6% for the Group 1 and Group 2 AID intervention arms, respectively. In Group 1, mean HbA1c decreased significantly from 9.1% ± 0.9% at baseline to 7.7% ± 1.0% in the AID arm (Δ of −1.4 ± 1.1%) and from 8.9% ± 1.1% at baseline to 8.2% ± 0.9% in the MDI arm (Δ of −0.6 ± 0.9%) the mean difference of −0.7% (−1.1% to −0.3%] (P = 0.0002) favored the AID intervention (**Table 3**). For the co-primary endpoint, there was a significant mean difference in %TBR <70 mg/dL in favor of the AID intervention for Group 2 (−4.8% [−6.4% to −3.1%], P < 0.0001) (**Table 3**). Given the rejection of the null hypotheses for the primary endpoints, secondary endpoint testing determined a significant mean difference in %TBR <70 mg/dL for Group 1 (−3.6% [−5.4% to −1.9%], P < 0.0001) that, also, favored AID intervention. The reduction in time spent below range for each group was 1.2 and 0.9 h/day, respectively, compared with the MDI control arm. For Group 2, mean HbA1c (7.1% ± 0.6%) remained stable in the AID arm and decreased by 0.1% in the MDI arm.

**Table 3:** Differences in primary and secondary endpoints between the AID intervention and control arm, stratified by baseline HbA1c.

	AID				MDI control				Difference (AID − MDI)	P
	N	Baseline	Study end	Δ	N	Baseline	Study end	Δ		
Primary endpoints										
Group 1: HbA1c, %	56	9.1 ± 0.9	7.7 ± 1.0	−1.4 ± 1.1	54	8.9 ± 1.1	8.2 ± 0.9	−0.6 ± 0.9	−0.7 [−1.1, −0.3]	0.0002^a^
Group 2: TBR<70 mg/dL, %	73	8.0 ± 6.8	2.8 ± 2.7	NA	69	8.6 ± 5.7	7.5 ± 6.1	NA	−4.8 [−6.4, −3.1]	<.0001^b^
Secondary endpoints										
Group 1: TBR<70 mg/dL, %	56	4.8 ± 4.4	1.9 ± 1.6	NA	54	4.4 ± 4.2	5.5 ± 5.9	NA	−3.6 [−5.4, −1.9]	<.0001^a^
Group 2: HbA1c, %	73	7.1 ± 0.6	7.1 ± 0.6	0.0 ± 0.6	69	7.1 ± 0.6	7.0 ± 0.7	−0.1 ± 0.5	0.1 [−0.1, 0.3]	0.0014^b^

Primary glycemic endpoints (superiority test) based on a baseline HbA1c of >8.0% [Group 1] or a baseline HbA1c of ≤8.0% [Group 2]. Comparative analyses (one-way ANOVA) are shown between the AID arm and control arm for either HbA1c or %TBR <70 mg/dL (<3.9 mmol/L) difference (baseline versus 6 months).

Data are presented as mean ± SD or mean [95% CI].

Time in AID was 79.6 ± 19.7% and 86.2 ± 13.4% for the Group 1 AID arm and Group 2 AID arm, respectively.

a Comparison of change in HbA1c between AID intervention and MDI control.

b Comparison of end-of-study %TBR <70 mg/dL (<3.9 mmol/L) between AID intervention and MDI control.

### 24-hour sensor glucose profiles

**Figure 2:**
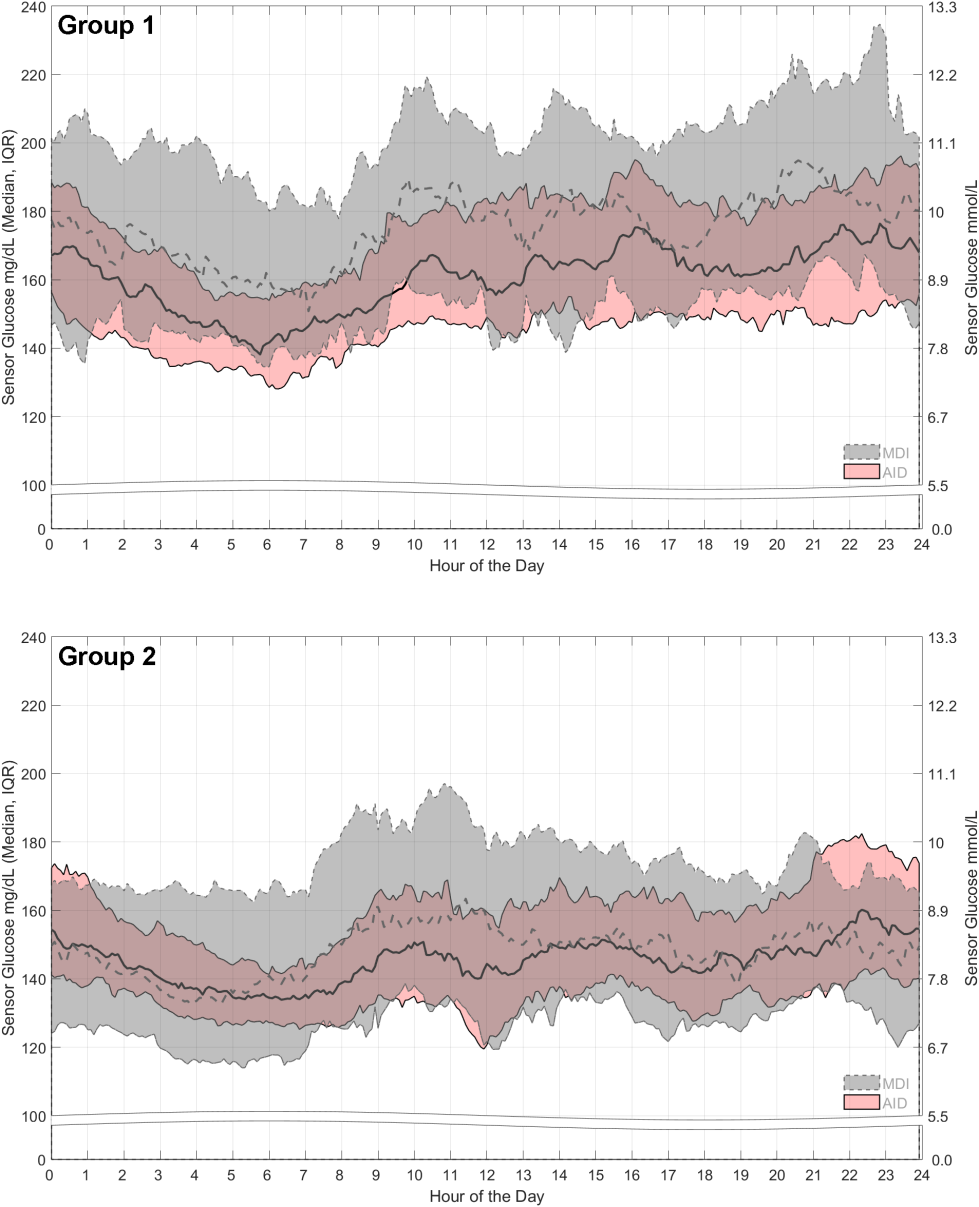
24-hour sensor glucose profiles of the AID intervention versus control, by baseline HbA1c group. The medians and interquartile ranges of the 24-h sensor glucose profiles after randomization to the AID intervention arm (pink band with solid lines) or MDI control arm (gray band with dashed lines) are shown by group. For both groups, sensor glucose data are based on the 2 weeks before the 6-month follow-up visit. The Group 1 AID intervention arm had N = 14 participants aged 2–17 years and N = 36 participants aged 18–80 years, and the MDI control arm had N = 8 aged 2–17 years and N = 30 aged 18–80 years. The Group 2 AID intervention arm had N = 19 participants aged 2–17 years and N = 46 participants aged 18–80 years, and the MDI control arm had N = 7 aged 2–17 years and N = 48 aged 18–80 years.

**Figure 3:**
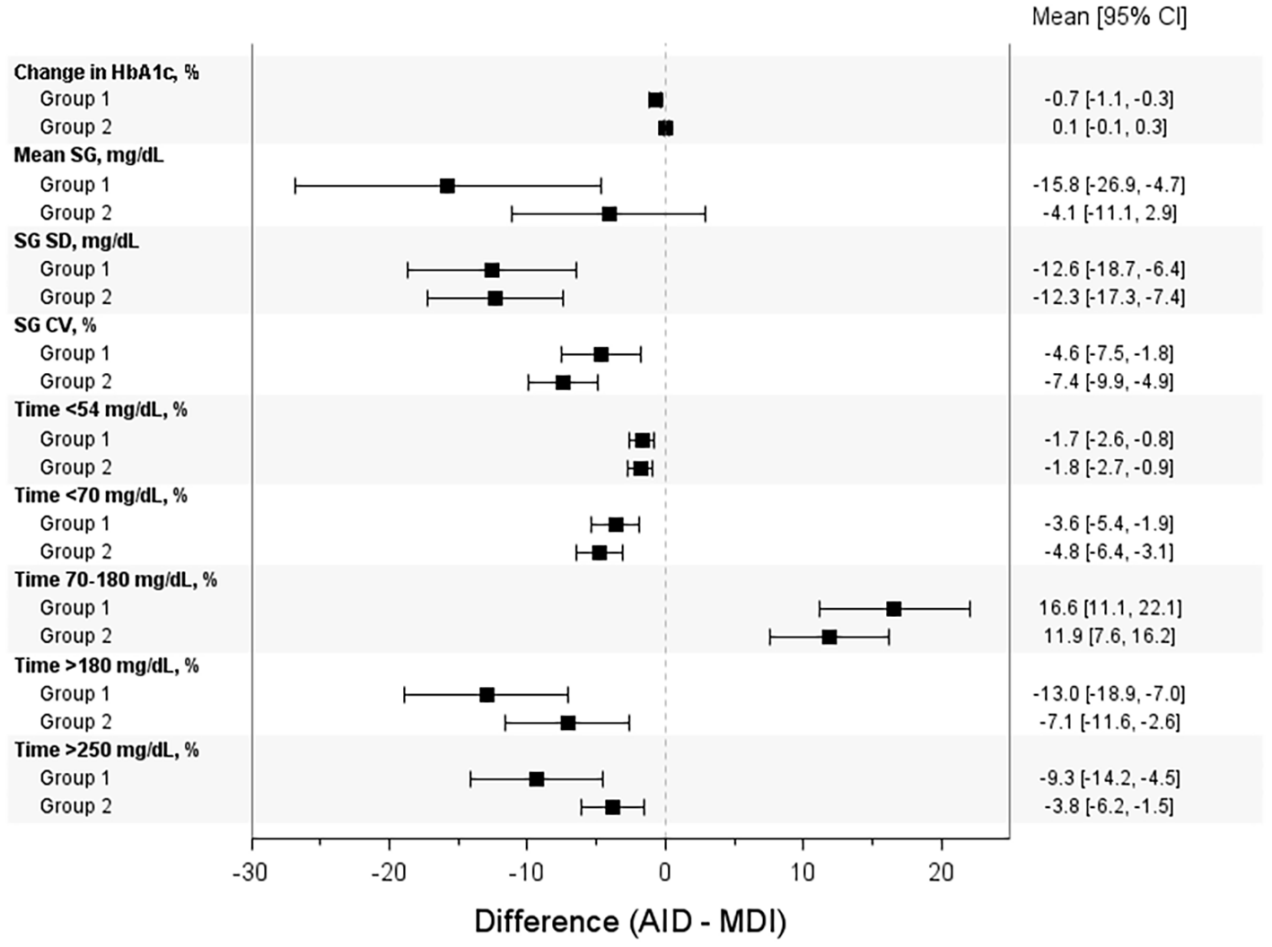
Differences in key glycemic endpoints between the AID intervention versus control, by baseline HbA1c group. The mean [95% CI] difference in change in HbA1c and additional CGM-derived metrics are shown by group, for the AID intervention arm versus MDI control arm.

The 24-h SG profiles are shown in **Figures 2** and **3**. The Group 1 median of SG for the AID intervention arm remained well below the median of SG for the MDI arm that had an upper interquartile range (IQR) spanning 180 to 230 mg/dL (10.0 to 12.8 mmol/L) across the 24-h day (**Figure 2**). In contrast, the AID intervention upper IQR spanned 150 to 190 mg/dL (8.3 to 10.6 mmol/L). For Group 2, the median of SG for the AID intervention arm closely matched that for the MDI arm during the late evening to early morning, and its IQR was nested within the MDI IQR (**Figure 3**). To add, the Group 2 AID IQR also appeared tighter than that observed for the Group 1 AID arm, especially during the early morning (2:00 AM–8:00 AM). Overall, the AID intervention appeared to provide an incrementally greater reduction in hyperglycemia for both groups.

### CGM-derived endpoints

The differences in key CGM-derived metrics (including mean SG, SD of SG, CV of SG, and %TAR >180 mg/dL) between AID intervention and control are illustrated in **Figure 3** and shown by group. The Forest plot demonstrates that all parameters were significantly improved with AID intervention except for change in HbA1c and mean SG in Group 2. The CGM-derived glycemic metrics for Group 1 and 2 AID interventions versus MDI control are described for participants aged 2–17 years (**Supplementary Materials S5**) and participants aged 18–80 years (**Supplementary Materials S6**).

### Participant-reported outcomes

**Table 4** shows that Group 1 of the AID intervention arm had a significantly higher end-of-study DTSQs and DTSQc total scores, when compared with those of the control arm (29.2 ± 6.4 versus 23.9 ± 6.4 [P = 0.0152] and 12.8 ± 6.3 versus 6.5 ± 7.1 [P = 0.0002], respectively). The same was observed regarding significantly reduced perceived frequency of hyperglycemia and hypoglycemia scores, in favor of AID intervention (**Table 4**). In contrast, the total DTSQs and DTSQc scores for the Group 2 AID intervention arm (26.4 ± 7.1 and 8.5 ± 8.4, respectively) were much lower and did not differ from those observed in the Group 2 control arm (25.8 ± 6.6 and 5.7 ± 7.0, respectively). Only the change in perceived frequency of hypoglycemia was significantly reduced for the Group 2 AID intervention arm, compared with control (P = 0.0055).

**Table 4:** Adult diabetes treatment satisfaction with the AID intervention and control arm, by baseline HbA1c group.

	AID			MDI control			Difference (AID − MDI)	P
	Baseline	Study end	Δ	Baseline	Study end	Δ		
Baseline HbA1c of >8.0% [Group 1]								
DTSQ(s), N	41	37	36	42	34	34	–	–
Total score^a^	23.6 ± 6.0	29.2 ± 6.4	5.1 ± 9.1	23.6 ± 5.3	23.9 ± 6.4	0.3 ± 6.7	4.8 (1.0, 8.6)	0.0152
Perceived frequency of hyperglycemia	3.7 ± 1.4	2.4 ± 1.4	−1.3 ± 1.8	3.6 ± 1.3	3.5 ± 1.4	−0.0 ± 1.8	−1.2 (−2.1, −0.4)	0.0052
Perceived frequency of hypoglycemia	2.3 ± 1.6	1.7 ± 1.1	−0.4 ± 1.7	1.7 ± 1.2	2.1 ± 1.3	0.6 ± 1.5	−1.0 (−1.8, −0.3)	0.0082
DTSQ(c), N	-	-	36	-	-	34	–	–
Total score^a^	NA	NA	12.8 ± 6.3	NA	NA	6.5 ± 7.1	6.3 (3.1, 9.5)	0.0002
Perceived frequency of hyperglycemia	NA	NA	−0.8 ± 1.8^b^	NA	NA	0.6 ± 1.4	−1.4 (−2.2, −0.6)	0.0005
Perceived frequency of hypoglycemia	NA	NA	−0.7 ± 1.6^b^	NA	NA	−0.0 ± 1.1	−0.7 (−1.3, −0.0)	0.0360
Baseline HbA1c of ≤8.0% [Group 2]								
DTSQ(s), N	53	45	45	61	57	56	–	–
Total score^a^	25.5 ± 6.8	26.4 ± 7.1	0.7 ± 10.2	25.8 ± 6.0	25.8 ± 6.6	0.5 ± 5.2	0.2 (−2.9, 3.4)	0.8828
Perceived frequency of hyperglycemia	3.0 ± 1.4	2.7 ± 1.6	−0.3 ± 1.9	3.1 ± 1.4	3.0 ± 1.5	−0.2 ± 1.8	−0.1 (−0.8, 0.7)	0.8493
Perceived frequency of hypoglycemia	2.5 ± 1.5	1.8 ± 1.3	−0.7 ± 1.7	2.3 ± 1.3	2.3 ± 1.2	0.0 ± 1.3	−0.7 (−1.3, −0.1)	0.0262
DTSQ(c), N	-	-	45	–	–	58	–	–
Total score^a^	NA	NA	8.5 ± 8.4	NA	NA	5.7 ± 7.0	2.7 (−0.3, 5.8)	0.0744
Perceived frequency of hyperglycemia	NA	NA	0.2 ± 1.9^c^	NA	NA	0.4 ± 1.4	−0.2 (−0.8, 0.5)	0.6253
Perceived frequency of hypoglycemia	NA	NA	−0.7 ± 1.6^c^	NA	NA	0.1 ± 1.3	−0.8 (−1.3, −0.2)	0.0055

^a^Total score includes items 1, 4–8 for adult version.

Data are presented as mean ± SD or mean (95% CI).

^b^N=37, ^c^N=46.

### Safety endpoints

Preestablished safety success criteria were met for both severe hypoglycemia and DKA. There was one DKA event (Group 1/adult participant) that occurred in the AID intervention arm (1.82 per 100 patient-years), and there were two severe hypoglycemic events (Group 2/adult participants) that occurred in the MDI control arm (3.52 per 100 patient-years). There were no SADEs, UADEs, or deaths in the trial.

## Discussion

This international, multicenter, randomized study of 6-month MM670G/770G system AID versus MDI (with or without CGM) evaluated a large population of adults and youth living with T1D and demonstrated the superiority of AID, in terms of clinically significant difference in change in HbA1c (−0.7%) for Group 1 (baseline HbA1c >8.0%) and difference in %TBR <70 mg/dL (−4.8%) for Group 2 (baseline HbA1c ≤8%), when compared with control. The study also demonstrated system safety across all participants, as event rates for DKA (N = 1, 1.82 per 100 patient-years) and severe hypoglycemia (N = 0) met preestablished success criteria for AID intervention and aligned closely with those observed during the MM670G AID versus CSII control RCT (6).

The present study also demonstrated improved treatment satisfaction with AID compared with MDI control. The differences between the AID intervention and control DTSQs and DTSQc scores were 4.8 (95%CI 1.0, 8.6) and 6.3 (95%CI 3.1, 9.5) respectively for Group 1, although only 0.2 (95%CI −2.9, 3.4) and 2.7 (95%CI −0.3, 5.8), respectively, for Group 2. The lower differences for Group 2 may have been due to some with a lower baseline HbA1c (and greater baseline glycemic control) having a higher satisfaction expectation that was unmet, which has been reported for a previous MiniMed™ 670G AID study (7). There may also have been an element of burden associated with trial protocol requirements for this group (8). Although a study effect could have positively impacted diabetes treatment satisfaction for the Group 1 control arm, given the clinically significant reduction in their HbA1c (−0.6%), their total DTSQs and perceived frequency of hyperglycemia and hypoglycemia scores were fairly similar from baseline to study end, suggesting benefits with AID in addition to improved glycemic control. Improved glycemic and psychosocial outcomes observed with AID intervention, in the present study, have substantial relevancy as they strengthen and support the well-being and quality of life of people living with T1D, factors that are often associated with AID therapies (9).

Similar benefits of lowered HbA1c and reduced time spent in hypoglycemia have been observed in pediatric and adult T1D, with another basal AID therapy. A single-arm, non-randomized follow-up extension trial of the Omnipod™ 5 system (Insulet Corporation, Acton, MA, USA) reported clinically significant reductions of −0.8% in the HbA1c of children (N = 108, aged 10.4 ± 2.1 years, baseline HbA1c of 7.7 ± 0.9 [5.8, 10.3]) and of −0.5% in the HbA1c of adolescents and adults (N = 114, aged 36.8 ± 14.0 years, baseline HbA1c of 7.2 ± 0.9% [5.2, 9.8]) at 6 months, compared with standard therapy (10). A clinically significant reduction in %TBR <70 mg/dL was also observed for the older participants, at 6 months. Although only 11.8% of youth and 16.7% of adults used MDI for standard therapy, glycemic improvements were reported for both age groups, regardless of baseline HbA1c.

The initialization of diabetes technology use after MDI therapy (11) or the addition of CGM to CSII therapy (12, 13) can significantly influence diabetes management behavior and markedly improve glycemic outcomes that are dependent on the extent of that technology use (14). The AID glycemic improvement in the present study, when compared with MDI outcomes, or with CSII for the other parallel RCT (6), strongly supports this. While MDI, with or without CGM, may serve as standard of care in developing regions due to lack of access or funding, optimized T1D glycemic control can be especially challenging or unattainable for youth or others living with significant dysglycemia or fear of hypoglycemia (15). In the present study, the overall group of participants (youth and adults) spent 77.6 ± 20.6% of time in closed loop, which appeared to differ for the younger participants (72.1 ± 22.7% [N = 16]) relative to the adults (79.6 ± 19.7% [N = 40]). Nevertheless, our exploratory subgroup analysis showed that AID intervention versus MDI control demonstrated a clinically significant increase in %TIR and a profound reduction in mean SG and %TAR for those in Group 1 and an appreciable reduction in %TBR <70 mg/dL for those in Group 2, for youth and adults.

Iterative advancements in diabetes technology including lower basal glucose targets and several active insulin time settings that personalize AID (4, 16–19) have been integral to improved HbA1c and CGM-derived glucose metrics (20–22), physiological normalization (23, 24), and better psychosocial outcomes (16, 18, 25). The advancement of AID technology from the first-in-class MiniMed™ 670G system with automated basal insulin to the MiniMed™ 780G system with automated correction insulin (available as often as every 5 min, as needed), in addition to automated basal insulin and lower glucose targets of 100 mg/dL (5.5 mmol/L) and 110 mg/dL (6.1 mmol/L) support this (26).

Strengths of the present study include the 6-month duration, the 1:1 randomized and parallel study design, and the evaluation of AID glycemic effectiveness with a standard therapy comparator (depending on national health care system, regulations, and reimbursement), in a large number of participants with T1D who represented different baseline glycemia (HbA1c ≤8.0% and HbA1c >8.0%). Study endpoints were supplemented with adult psychosocial outcomes and exploratory assessment of CGM-derived outcomes for the overall group and by age group.

A limitation of the study was the low percentage of individuals from underrepresented or minority groups. For example, compared with United States 2019–2021 estimates for adults with diabetes (27), Native American (0.6% versus 13.6%), Asian (0.8% versus 9.1%), non-Hispanic Black (2.8% versus 12.1%), and Hispanic (5.5% versus 11.7%) groups were underrepresented in the overall cohort. This introduces some bias in the observed study outcomes, as the enrolled participants most likely experience substantial differences in healthcare and social determinants of health. This disproportionate inclusion of diverse groups may limit the generalizability of study findings to a larger and diverse type 1 diabetes population. A second limitation is that not all participants in the MDI control group used CGM, and the study did not include a separate comparison between MDI primary and secondary endpoints with and without CGM use relative to AID intervention. Significantly improved outcomes with AID may, in part, have been due to BGM use with MDI, especially if inconsistent, as the T1D Exchange has demonstrated that BGM frequency is inversely proportional to suboptimal HbA1c (28) and CGM use is associated with better glycemic control achievement (29). In addition, only the participant-reported psychosocial outcomes of adults were analyzed.

In conclusion, this large, international, multicenter RCT study further demonstrates safe and significant HbA1c and %TBR <70 mg/dL reduction in T1D that favors MiniMed™ 670G/770G AID versus MDI with or without CGM, in addition to AID-improved treatment satisfaction.

## Adult and Pediatric MiniMed™ HCL Outcomes 6-month Randomized Controlled Trial: HCL versus MDI Control Study Group

Atlanta Diabetes Associates (Atlanta, Georgia, USA): Bruce W. Bode, MD, FACE. Azienda Ospedaliera Spedali Civili di Brescia (Brescia, Italy): Angela Girelli, MD and Cimino Elena, MD. Barbara Davis Center for Diabetes (Aurora, Colorado, USA): Satish K. Garg, MD. Institute of Metabolic Science and Department of Medicine, University of Cambridge (Cambridge, United Kingdom): Mark Evans, MD, Sarah Donald, Helen Brown, and Jane Baillie. Sutter Children’s Center and Sutter Medical Group (Sacramento, California, USA): Gnanagurudasan Prakasam, MD, MRCP, MHA. CHEO Research Institute (Ottawa, Ontario, Canada): Margaret L. Lawson, MD, MSc, MHSc, FRCP; Caroline Zuijdwijk, MD; Elizabeth Stevens RD, CDE; and Brenda J. Bradley RN. Diabetes and Glandular Disease Clinic, P.A. (San Antonio, Texas, USA): Mark S. Kipnes, MD. Diabeteszentrum für Kinder und Jugendliche, Kinder- und Jugendkrankenhaus Auf der Bult (Hannover, Germany): Prof. Thomas Danne, MD. Diablo Diabetes Center (Walnut Creek, California, USA): Mark P. Christiansen, MD. Endocrine Research Solutions (Roswell, Georgia, USA): John “Chip” H. Reed, MD; Ellen Medved, FNP-C, CDE; Jessica Tapia, CMA, CCRC; and Tabby Sapp, CMA, CRC. Grunberger Diabetes and Endocrinology (Bloomfield Hills, Michigan, USA): George Grunberger, MD, FACP, MACE and Elizabeth Shimoura, MS. HCL- Diab-eCare (Lyon, France): Charles Thivolet, MD and Sylvie Villar Fimbel, MD. Hôpital Necker Enfants Malades (Paris, France): Jacques Beltrand, MD. Department of Endocrinology and Nutrition, Hospital Universitario Virgen Macarena, Instituto de Biomedicina de Sevilla (Sevilla, Spain): María Asunción Martinez-Brocca, MD, PhD and Noelia Gros Herguido, MD. Indiana University School of Medicine (Indianapolis, Indiana, USA): Linda A. DiMeglio, MD, MPH. Iowa Diabetes Research (West Des Moines, Iowa, USA): Anuj Bhargava, MD, MBA, CDCES, FACP, FACE and Lisa Borg, CMA, CCRC. Mayo Clinic Rochester (Rochester, Minnesota, USA): Yogish C. Kudva, MBBS; Donna Desjardins, APRN; Vinaya Simha, MD; Shelly K. McCrady Spitzer, MSN; Corey Kurek, BSN; and Shafaq Rizvi, MB, BS. Medtronic Diabetes (Northridge, California, USA): Scott W. Lee, MD (current affiliation, Loma Linda University, Division of Endocrinology, Diabetes & Metabolism, San Diego, California, USA); Caroline Yovanovich, PhD; John J. Shin, PhD, MBA; Toni L. Cordero, PhD, Jennifer J.F. McVean, MD and Robert A.Vigersky, MD. New Zealand Clinical Research (Christchurch, New Zealand): Assoc. Prof. Martin I. de Bock, FRACP, PhD. Park Nicollet International Diabetes Center/Health Partners Institute (Minneapolis, Minnesota, USA): Anders L. Carlson, MD; Amy B. Criego, MD, MS; Richard M. Bergenstal, MD; Thomas W. Martens, MD, FACP; Diane Whipple, RDN, BSN, CCRC, CDCES; and Lindsey Smith, RDN, LD, CDCES. Rocky Mountain Diabetes & Osteoporosis Center (Idaho Falls, Idaho, USA): David R. Liljenquist, MD. Sanford Research (Sioux Falls, South Dakota, USA): Kurt J. Griffin, PhD, MD (current affiliation, Center for Interventional Immunology, Benaroya Research Institute, Seattle, Washington, USA); John Shelso, MD; and Alaa Al Nofal, MD. Sansum Diabetes Research Institute (Santa Barbara, California, USA): Jordan E. Pinsker, MD. Scripps Whittier Diabetes Institute (La Jolla, California, USA): Athena Philis-Tsimikas, MD; Kelley Barczi, RN, CDCES; and Rosario Rosal, RN. Stanford University School of Medicine (Stanford, California, USA); Bruce A. Buckingham, MD; Marissa Town, RN, BSN, CDCES; and Christine Weir, BS. SUNY Upstate Medical University (Syracuse, New York, USA): Ruth S. Weinstock, MD, PhD; David W. Hansen, MD; and Suzan Bzdick RN, CDCES, CCRC. Texas Diabetes and Endocrinology (Austin, Texas, USA): Luis Casaubon, MD. School of Medicine, Department of Medical Sciences, Örebro University (Örebro, Sweden): Prof. Johan H. Jendle, MD, PhD. University of Michigan (Ann Arbor, Michigan, USA): Rodica Pop-Busui, MD, PhD. University of Washington Medical Center (Seattle, Washington, USA): Irl B. Hirsch, MD, MACP. Health New Zealand - Southern (Dunedin, New Zealand): Prof. Benjamin J. Wheeler, FRACP, PhD and Alisa Boucsein, PhD. Washington University - Saint Louis (St. Louis, Missouri, USA): Janet B. McGill, MD, MA, FACE, FACP; Maamoun Salam, MD; and Carol Recklein, BSN, CDE. Westminster endocrine & Diabetes research (New westminster, Canada): B. Anne Priestman, MD, FRCPC.

## Data Availability

The original contributions presented in the study are included in the article/**Supplementary Material**. Further inquiries can be directed to the corresponding author.
