# Supplementary material for: Automated basal insulin delivery versus multiple daily injections in type 1 diabetes: results from a randomized parallel controlled trial
Source: Front Endocrinol (Lausanne). 2025 Dec 19;16:1716587. doi: 10.3389/fendo.2025.1716587 (PMC12757873; doi:10.3389/fendo.2025.1716587)
Supplement: Supplementary file 1 [file DataSheet1.pdf]

Supplementary Materials (On-line Only)

S1. Inclusion and exclusion criteria

**Inclusion criteria**
1. Participant is aged 2-80 years at time of screening<br>2. Participant is aged 2-21 years and determined by the investigator to have the appropriate, requisite support (family, caregiver or social network) to successfully participate in this study<br>3. Participant with a minimum daily insulin requirement (total daily dose) ≥8 units/day<br>4. Participant who is determined by the investigator to be psychologically sound in order to successfully participate in this study<br>5. Participant who has been diagnosed with type 1 diabetes for at least three months<br>Note: Determination of classification for diabetes will be based on *American Diabetes Association Clinical Practice Guidelines* accounting for several patient characteristics including age of onset, weight or BMI, history of diabetic ketoacidosis, and history of therapy management, if available in the medical records<br>6. Participant must be on one of the following management therapies:<br>• Multiple daily injections defined by use of rapid analogue with meals and approved long-acting analogue (e.g., detemir or glargine) with or without CGM<br>7. Participant who is willing to perform ≥ 4 finger stick blood glucose measurements daily<br>8. Participant who is willing to perform required study procedures<br>9. Participant who is willing to wear the system continuously throughout the study for at least 80% of the time.<br>10. Participant who is willing to upload data at least weekly from the study pump/meter, have Internet access and a computer system that meets the requirements for uploading the study pump/meter for data collection<br>11. Participant who is willing to use the study glucose meter system (i.e., along with study meter strips)<br>12. Participant with celiac disease that has been adequately treated as determined by the investigator<br>13. Participant with a diagnosis of myocardial infarction, unstable angina, coronary artery bypass surgery, coronary artery stenting, transient ischemic attack, cerebrovascular accident, angina, congestive heart failure, ventricular rhythm disturbances or thromboembolic disease, within 1 year of screening, who has the consent of the Investigator<br>14. Participant who is willing to take one of the following insulins and can financially afford to use either of the 2 insulin preparations throughout the course of the study (i.e., co-payments for insulin with insurance or able to pay full amount)<br>a. Insulin lispro (Eli Lilly) or Insulin aspart (Novo Nordisk A/S)
**Exclusion criteria**
1. Participant has participated in any closed loop study in the past<br>2. Participant is unable to tolerate tape adhesive in the area of sensor placement<br>3. Participant has any unresolved adverse skin condition in the area of sensor placement (e.g., psoriasis, rash, *Staphylococcus* infection) or area of infusion set placement<br>4. Women of child-bearing potential who have a positive pregnancy test at screening or plan to become pregnant during the course of the study<br>5. Participant is being treated for hyperthyroidism at time of screening<br>6. Participant has an abnormality (out of reference range) in thyroid-stimulating hormone (TSH) at time of screening visit. TSH is not required for participants 2-13 years of age

7. Participant has taken any oral, injectable, or IV glucocorticoids within 8 weeks from time of screening visit, or plans to take any oral, injectable, or IV glucocorticoids during the course of the study
8. Participant is actively participating in an investigational study (drug or device) wherein he/she has received treatment from an investigational study drug or investigational study device in the last 2 weeks
9. Participant is currently abusing illicit drugs or marijuana
10. Participant is currently abusing prescription drugs
11. Participant is currently abusing alcohol
12. Participant is using pramlintide, SGLT2 inhibitors, GLP agonists, biguanides, DPP-4 inhibitors or sulfonylureas at time of screening
13. Participant is using hydroxyurea at the time of screening or plans to use it during the study
14. Participant has a history of visual impairment which would not allow participant to participate in the study and perform all study procedures safely, as determined by the investigator
15. Participant has a sickle cell disease, hemoglobinopathy; or has received red blood cell transfusion or erythropoietin within 3 months prior to time of screening
16. Participant plans to receive red blood cell transfusion or erythropoietin over the course of study participation
17. Participant has been diagnosed with current moderate to severe eating disorder such as anorexia or bulimia
18. Participant has been diagnosed with chronic kidney disease requiring dialysis or resulting in chronic anemia
19. Participants who are currently being actively treated for cancer
20. Participant who is designated as a research staff member for this study

S2. Study visit schedule

<table>
<tr><td colspan="2">Screening and run-in period (Completed within 60 days)</td></tr>
<tr><td colspan="2">• Visit 1: Consent and screening
    o Administer questionnaires
• Visit 2: Begin run-in period
    o Laboratory A1C
    o Masked CGM for 2 Weeks
• Visit 3: Randomization
    o AID intervention arm – Start MiniMed™ 670G/770G pump without sensor
    o MDI control arm – Continue with MDI</td></tr>
<tr><td colspan="2">Study period</td></tr>
<tr><td>AID arm
• Visit 4: Start CGM
• Visit 4A: Follow up telephone visit
• Visit 5: Follow up office visit
    o Auto Mode started
    o Investigator sets carbohydrate to insulin ratios, active insulin time, and basal rates for open loop periods
• Visit 6A: Follow-up telephone or office visit
• Visit 6B: Follow-up telephone or office visit
• Visit 6C: Follow-up telephone visit
• Visit 7A: Follow up office visit
• Visit 7B: Follow-up telephone visit
• Visit 7C: Follow up office visit
• Visit 7D: Follow-up telephone visit
• Visit 8: Follow up office visit
• Visit 9: Follow up office visit - 180 days after randomization
    o Administer questionnaires
    o End of study period</td>
<td>MDI control arm
• Visit 4: Day 0
• Visit 5: Follow up office visit
• Visit 6A: Follow up telephone visit
• Visit 6B: Follow up telephone visit
• Visit 6C: Follow up telephone visit
• Visit 6D: Follow up office Visit
• Visit 6E: Follow-up telephone visit
• Visit 7: Follow up office visit
    o Masked CGM start
    o 14 days of sensor wear before 90-day study period visit.
• Visit 8A: Follow up office visit – 90 Days after Randomization
    o Masked CGM return
• Visit 8B: Follow up office visit - 166 days after Randomization
    o Masked CGM start
    o 14 days of sensor wear before 180-day study period visit.
• Visit 9: Follow up office visit – 180 days after Randomization
    o Masked CGM return
    o End of study period</td></tr>
</table>

S3. Study disposition

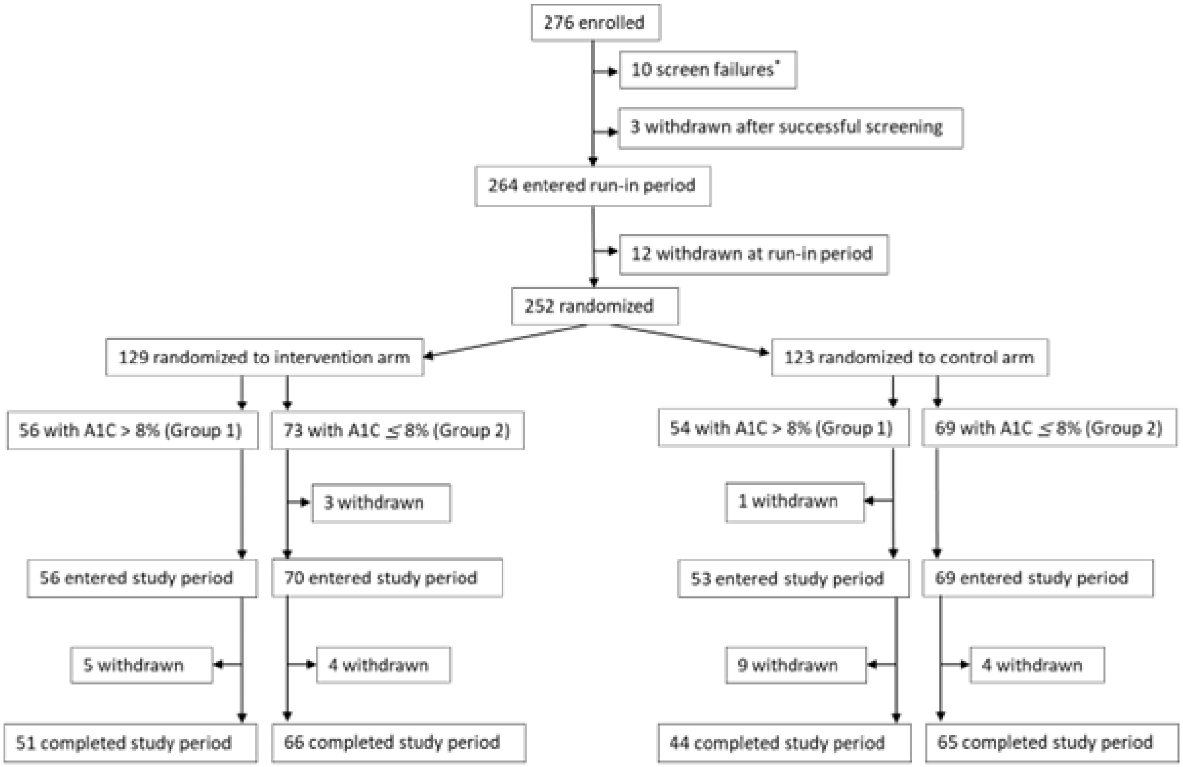

*N=1 was re-screened

S4. Overall group demographics and baseline characteristics

	OVERALL	*U.S.*	*EUROPE*	*CANADA*	*NEW ZEALAND*
	(n=252)	(n=165)	(n=70)	(n=15)	(n=2)
Age, years					
Mean (SD)	36.0 (19.6)	37.4 (20.0)	36.3 (17.0)	22.5 (21.8)	8.5 (3.5)
Range (min, max)	4.0, 76.0	4.0, 76.0	7.0, 76.0	6.0, 72.0	6.0, 11.0
Sex, n (%)					
Male	171 (67.9%)	113 (68.5%)	47 (67.1%)	11 (73.3%)	0 (0.0%)
Female	81 (32.1%)	52 (31.5%)	23 (32.9%)	4 (26.7%)	2 (100.0%)
Baseline A1C, %					
Mean (SD)	7.9 (1.2)	8.1 (1.4)	7.7 (0.9)	7.6 (0.8)	7.5 (1.1)
Range (min, max)	5.5, 13.1	5.5, 13.1	5.6, 9.7	6.0, 8.8	6.7, 8.2
Baseline A1C, mmol/mol					
Mean	63.2 (13.6)	64.7 (15.0)	60.6 (10.2)	59.3 (9.3)	57.9 (11.6)
Range (min, max)	36.6, 119.7	36.6, 119.7	37.7, 82.5	42.1, 72.7	49.7, 66.1
Diabetes history, years					
Mean	17.1 (14.2)	17.6 (13.7)	19.2 (15.1)	4.6 (6.4)	1.0 (0.0)
Range (min, max)	0.0, 61.0	0.0, 61.0	0.0, 60.0	0.0, 19.0	1.0, 1.0
Race					
White	212 (84.1%)	151 (91.5%)	47 (67.1%)	13 (86.7%)	1 (50.0%)
Asian	2 (0.8%)	2 (1.2%)	0 (0.0%)	0 (0.0%)	0 (0.0%)
Indigenous/First Nations	1 (0.4%)	1 (0.6%)	0 (0.0%)	0 (0.0%)	0 (0.0%)
Asian; White	1 (0.4%)	0 (0.0%)	0 (0.0%)	1 (6.7%)	0 (0.0%)
Black African	7 (2.8%)	7 (4.2%)	0 (0.0%)	0 (0.0%)	0 (0.0%)
Black African; White	1 (0.4%)	1 (0.6%)	0 (0.0%)	0 (0.0%)	0 (0.0%)
Not reportable per local laws	22 (8.7%)	0 (0.0%)	22 (31.4%)	0 (0.0%)	0 (0.0%)
Other	5 (2.0%)	3 (1.8%)	1 (1.4%)	1 (6.7%)	0 (0.0%)
White; Other	1 (0.4%)	0 (0.0%)	0 (0.0%)	0 (0.0%)	1 (50.0%)
Ethnicity					
Non-Hispanic/Latino	208 (82.5%)	156 (94.5%)	35 (50.0%)	15 (100.0%)	2 (100.0%)
Hispanic/Latino	22 (8.7%)	9 (5.5%)	13 (18.6%)	0 (0.0%)	0 (0.0%)
Not reportable per local laws	21 (8.3%)	0 (0.0%)	21 (30.0%)	0 (0.0%)	0 (0.0%)
Not reported	1 (0.4%)	0 (0.0%)	1 (1.4%)	0 (0.0%)	0 (0.0%)

Data are shown as mean (SD) or n (%).
A1C, glycated hemoglobin; AID, automated insulin delivery; SD, standard deviation.

S5. Glycemic outcomes for randomized participants aged 2-17 years, by baseline A1C group

	**Group 1 (Baseline A1C >8.0%)**				**Group 2 (Baseline A1C ≤8.0%)**			
	**AID**		**MDI**		**AID**		**MDI**	
	**Baseline**	**Study End**	**Baseline**	**Study End**	**Baseline**	**Study End**	**Baseline**	**Study End**
	**n=16**		**n=12**		**n=21**		**n=9**	
**Mean SG, mg/dL**	200.7 (32.6)	176.0 (21.4)	198.5 (26.2)	188.8 (32.2)	152.3 (28.2)	156.5 (20.3)	141.0 (18.9)	153.8 (33.6)
**SD of SG, mg/dL**	80.2 (16.0)	68.7 (14.4)	76.4 (10.6)	74.2 (16.9)	61.0 (17.7)	55.1 (11.6)	63.1 (16.8)	75.1 (31.0)
**CV of SG, %**	40.3 (7.3)	38.8 (4.8)	39.1 (7.9)	40.0 (9.6)	39.5 (8.5)	35.0 (4.7)	44.5 (9.3)	47.9 (12.5)
**Percentage of time spent in SG ranges, mg/dL**								
**<54 mg/dL**	1.7 (2.8)	0.5 (0.7)	1.2 (1.4)	1.5 (2.2)	1.3 (1.4)	0.6 (0.7)	3.0 (3.2)	5.5 (5.4)
**<70 mg/dL**	4.7 (4.1)	2.0 (1.9)	3.8 (3.9)	4.8 (4.2)	5.9 (4.2)	2.2 (2.2)	11.4 (6.6)	13.1 (8.7)
**70-180 mg/dL**	38.9 (12.8)	58.4 (9.2)	40.0 (10.9)	43.7 (15.2)	64.3 (14.4)	69.0 (11.8)	63.4 (12.8)	56.3 (18.7)
**>180 mg/dL**	56.4 (14.6)	39.6 (10.1)	56.2 (12.5)	51.6 (16.3)	29.8 (16.2)	28.9 (12.2)	25.2 (11.2)	30.6 (18.4)
**>250 mg/dL**	27.1 (15.0)	15.7 (8.9)	26.5 (12.2)	22.9 (18.3)	10.0 (7.2)	8.0 (7.1)	7.3 (6.7)	13.9 (12.0)

Data are shown as mean (SD).

Time in AID was 72.1±22.7% and 83.3±14.2% for the Group 1 AID arm and Group 2 AID arm, respectively.

SG, sensor glucose; SD, standard deviation; CV, coefficient of variation of sensor glucose; N, number of randomized participants.

S6. Glycemic outcomes for randomized participants aged 18-80 years, by baseline A1C group

	**Group 1 (Baseline A1C >8.0%)**				**Group 2 (Baseline A1C ≤8.0%)**			
	**AID**		**MDI**		**AID**		**MDI**	
	**Baseline**	**Study End**	**Baseline**	**Study End**	**Baseline**	**Study End**	**Baseline**	**Study End**
	**n=40**		**n=42**		**n=52**		**n=60**	
**Mean SG, mg/dL**	190.2 (28.3)	159.8 (14.3)	181.0 (26.9)	177.8 (34.3)	144.4 (20.6)	144.9 (11.3)	145.8 (19.0)	152.2 (21.7)
**SD of SG, mg/dL**	76.6 (14.8)	53.6 (11.1)	68.6 (13.9)	69.4 (14.9)	56.9 (13.5)	46.7 (9.6)	57.7 (12.7)	59.5 (12.1)
**CV of SG, %**	40.6 (7.4)	33.3 (4.9)	38.2 (7.4)	39.3 (7.8)	39.5 (9.0)	32.1 (5.5)	39.6 (7.3)	39.3 (7.0)
**Percentage of time spent in SG ranges, mg/dL**								
**<54 mg/dL**	1.7 (2.4)	0.4 (0.5)	1.5 (2.4)	2.2 (3.3)	3.4 (4.1)	0.8 (1.1)	2.9 (3.2)	2.1 (2.9)
**<70 mg/dL**	4.9 (4.6)	1.8 (1.5)	4.5 (4.3)	5.7 (6.3)	8.7 (7.4)	3.0 (2.9)	8.1 (5.5)	6.7 (5.2)
**70-180 mg/dL**	44.2 (14.5)	68.5 (10.2)	48.0 (13.8)	50.5 (15.0)	66.0 (13.2)	76.6 (8.8)	65.8 (11.8)	63.4 (12.7)
**>180 mg/dL**	50.9 (15.8)	29.7 (10.3)	47.4 (15.5)	43.9 (17.0)	25.3 (13.5)	20.4 (8.3)	26.1 (12.1)	29.9 (13.8)
**>250 mg/dL**	23.5 (12.3)	7.4 (5.5)	17.4 (11.0)	18.0 (13.9)	6.4 (5.1)	3.5 (3.1)	6.3 (5.4)	7.9 (6.8)

Data are shown as mean (SD).

Time in AID was 79.6±19.7% and 86.2±13.4% for the Group 1 AID arm and Group 2 AID arm, respectively.

SG, sensor glucose; SD, standard deviation; CV, coefficient of variation of sensor glucose; N, number of randomized participants.
